# Patient-derived organoids predict chemotherapy response of locally advanced gastric cancer

**DOI:** 10.1371/journal.pone.0339416

**Published:** 2026-03-09

**Authors:** Miao Huang, Jiahui Chu, Wenbin Yu, Liliang Dou, Qiushi Wang, Fan Jiang, Meng Wei, Xiaohan Cui, Wen Zhao, Jianyuan Zhou, Song Li, Lian Liu

**Affiliations:** 1 Department of Medical Oncology, Qilu Hospital, Cheeloo College of Medicine, Shandong University, Jinan, China; 2 Institute of Marine Science and Technology, Shandong University, Qingdao, China; 3 Department of pharmacy, Qilu Hospital, Cheeloo College of Medicine, Shandong University, Jinan, China; 4 Department of General Surgery, Qilu Hospital, Cheeloo College of Medicine, Shandong University, Jinan, China; QUT Health: Queensland University of Technology Faculty of Health, AUSTRALIA

## Abstract

The efficacy of standard adjuvant chemotherapy for locally advanced gastric cancer (GC) remains suboptimal, particularly in patients with signet-ring cell carcinoma (SRCC). Urgent demand exists for reliable preclinical models to predict therapeutic responses, and *in vitro* drug sensitivity testing using patient-derived organoids (PDOs) has emerged as a promising platform. In this study, PDOs were established from patients with locally advanced GC and analyzed via next-generation sequencing (NGS) and pharmacotyping. Seventeen GC PDOs were successfully generated, achieving a success rate of 63%. These PDOs closely recapitulated the histopathological and genetic features of their parental tumors. Drug sensitivity tests revealed subtype-specific response patterns: PDOs derived from SRCC were sensitive to epirubicin and paclitaxel but resistant to 5-fluorouracil (5-FU) and oxaliplatin. In contrast, non-SRCC PDOs demonstrated robust sensitivity to paclitaxel, epirubicin, and oxaliplatin. Among all tested drugs, paclitaxel showed the highest tumor-inhibitory efficacy in both subtypes. Furthermore, non-SRCC PDOs were significantly more sensitive to 5-FU and oxaliplatin than SRCC PDOs. *Ex vivo* pharmacotyping of PDOs accurately predicted clinical therapeutic responses in GC patients, with a sensitivity of 85.7%, specificity of 100%, and accuracy of 90.9%. Notably, patients whose PDOs were drug-sensitive *in vitro* had significantly longer disease-free survival than those whose PDOs were drug-resistant (*P* = 0.044). These findings highlight the potential of GC PDOs as reliable preclinical models that faithfully recapitulate tumor biology and therapeutic responses, thereby providing a valuable tool for predicting individualized treatment outcomes and advancing precision oncology for GC.

## Introduction

Gastric cancer (GC) is the fifth most common cancer and the fourth leading cause of cancer-related mortality worldwide [1]. In China, approximately 70% of GC patients are diagnosed at a locally advanced stage. Notably, survival outcomes for stage III GC vary markedly, with 5-year survival rates decreasing from 61–63% for stage IIIA to 30–35% for stage IIIC [2]. Therefore, improving the success rate of R0 resection (R0 resection is defined as the complete excision of all tumor tissue with histologically negative margins, indicating the absence of residual cancer cells at the resected edges) and enhancing postoperative survival are essential strategies to increase the overall survival (OS) of GC patients.

Most existing studies have focused on the timing and implementation of standardized perioperative chemotherapy regimens in the general population [3–5], whereas few have explored personalized strategies tailored to specific pathological subtypes or patient characteristics [6,7]. Standard adjuvant chemotherapy typically consists of either S-1 monotherapy (a combination of tegafur, gimeracil, and oteracil) or a regimen combining oxaliplatin with capecitabine [4,8]. However, the marked pathological heterogeneity of GC significantly limits treatment efficacy, resulting in suboptimal outcomes, with a 3-year disease-free survival (DFS) rate of only 50–60% [5]. This underscores the need for drug screening studies in the postoperative setting to identify the most effective chemotherapeutic agents for individual patients.

A variety of preclinical drug screening models have been developed, including tumor cell lines [9], three-dimensional (3D) tumor cell spheres cultured *in vitro* [10], patient-derived xenografts (PDXs) [11], patient-derived explants (PDEs) [12], patient-derived tumor fragments (PDTFs) [13], and patient-derived organoids (PDOs) [14]. Tumor cell lines and *in vitro* 3D-cultured tumor cell spheres often lose the genetic heterogeneity of the original tumors due to clonal selection [9,10]. Although PDXs preserve genetic heterogeneity of the original tumor, their utility is limited by high costs, low engraftment efficiency, and prolonged establishment time [11]. PDEs [12] and PDTFs [13] are relatively easy to establish and preserve the tumor immune microenvironment over the short term, but they have limited passaging capacity and are unsuitable for high-throughput drug screening. In contrast, recent advances in tumor PDO culture techniques have enabled the generation of self-renewing, tumor stem cell-derived models capable of self-organization in 3D *in vitro* culture. These PDOs retain the morphological and genetic characteristics of the parental tissue [14–16]. Compared with other preclinical models, PDOs offer several distinct advantages, including rapid establishment, high cellular viability, robust passaging capacity, relatively low cost, and compatibility with high-throughput screening platforms [16,17]. Moreover, multiple studies have shown that PDOs can closely mimic individual patient responses to therapy, underscoring their potential as a powerful platform for guiding precision medicine [14,15].

In this study, we established PDOs from resected tissues or biopsy specimens obtained from patients with locally advanced GC. These PDOs faithfully recapitulated the histopathological and genetic features of the parental tumors. Importantly, our assessment of the concordance between clinical chemotherapy responses and PDO drug-sensitivity profiles provides robust evidence that PDO models can serve as a translational tool to guide personalized adjuvant therapy, thereby bridging the gap between preclinical testing and precision oncology in GC.

## Materials and methods

### Patients and sample collection

From September to December 2022, a total of 27 GC tissue specimens were collected from patients who underwent resection or biopsy at Qilu Hospital of Shandong University. Patients with a history of other malignancies, metastatic lesions, or unknown primary tumor were excluded. This study was approved by the Scientific Research Ethics Committee of Qilu Hospital of Shandong University (approval number: KYLL-202205-021-2), and written informed consent was obtained from all participants.

### Establishment and culture of GC PDOs

Obtained GC tissue samples were suspended in transport medium and transported to the laboratory at 4°C [13]. One-third of the high-viability GC tissue was used for organoid culture, one-third was snap-frozen in liquid nitrogen for whole-exome sequencing (WES), and the remaining tissue was fixed in 4% paraformaldehyde for hematoxylin-eosin (H&E) and immunohistochemistry (IHC) staining. GC tissues were washed 3 times with ice-cold phosphate-buffered saline (PBS, Aoqing Biotechnology, Beijing, China) containing 100 µg/mL primocin (InvivoGen, Hong Kong, China) and 0.1% bovine serum albumin (BSA, Solarbio, Beijing, China), then minced into fragments <0.5 cm in diameter. The tissue fragments were incubated in digestion buffer DMEM/F12 (KeyGEN Biotechnology, Jiangsu, China) containing 100 µg/mL primocin, 2 mg/mL collagenase IV (Sigma-Aldrich, St. Louis, MO, USA), 200 µg/mL pulmozyme (Sigma-Aldrich), and 0.1 mg/mL hyaluronidase (Solarbio) for 30–60 min at 37 °C. Tissue debris was removed by filtering through a 70 μm cell strainer. Cell clusters were washed twice by centrifugation at 1000 rpm for 5 min, resuspended in pre-cooled Matrigel (Abwbio, Shanghai, China), and seeded in a pre-heated 24-well plate as 50 µL droplets per well. After droplet solidification, 500 µL of GC organoid complete medium (1x GC organoid basal medium (BioGenousTM, Suzhou, China), 1x GC organoid supplement B (BioGenousTM), 1x GC organoid supplement C (BioGenousTM) and 10 µM Y-27632 (MCE, New Jersey, USA)) was added to each well. PDOs were cultured at 37 °C in a 5% CO₂ incubator with medium replaced every 2–3 days.

Organoid cultures were passaged every 2 weeks with a split ratio of 1:2–1:3. Matrigel was digested using harvest solution (R&D system, Minnesota, USA), and PDOs were incubated in TrypLE (Thermo Fisher Scientific, Waltham, MA, USA) at 37 °C for 3–5 min. The cell suspension was re-embedded in Matrigel. Cryopreservation and recovery of PDOs were performed as previously described [18].

### H&E and IHC staining of GC PDOs and parental tumor tissues

GC PDOs and their parental tumor tissues were fixed in 4% paraformaldehyde at 4 °C for 24 h, dehydrated through a graded ethanol series, cleared with xylene, and embedded in paraffin. PDOs were sectioned into 4-µm-thick slices and mounted onto glass slides. Hematoxylin-eosin (H&E) and immunohistochemistry (IHC) staining were performed according to a standard protocol [19]. The primary antibodies used for IHC included Ki67 (clone SP6, 1:600 dilution, Servicebio, Wuhan, China), CK7 (clone 7D8G8, 1:600 dilution, Servicebio, Wuhan, China), CDX2 (clone 4H2A8, 1:1500 dilution, Servicebio, Wuhan, China), and CK20 (clone SP33, 1:1500 dilution, Servicebio, Wuhan, China).

### WES analysis of GC PDOs and parental tumor tissues

Genomic DNA was extracted using the Magnetic Universal Genomic DNA Kit (TIANGEN, Beijing, China). PE150 paired-end sequencing was performed on the Illumina platform. Somatic single nucleotide variants (SNVs) and insertion-deletions (InDels) were identified using MuTect and Strelka, respectively. Somatic copy-number variations (CNVs) were detected with Control-FREEC. All somatic variants were annotated using ANNOVAR software [20]. The MuSiC software was employed to identify significantly mutated genes (SMGs) by integrating somatic SNVs and InDels [21]. A convolution test (CT) was performed using all or a subset of tumor samples as the background, and genes with a Q-value < 0.2 were retained as SMGs. Tumor mutational burden (TMB) was calculated based on somatic nonsynonymous mutations (SNVs/InDels) identified in tumor tissues. Variants with low sequencing depth (WES < 20 ×, WGS < 10×) were excluded, and only mutations with a variant allele frequency (VAF) ≥5% were retained. Low-frequency variants (AF < 1%) present in the 1000 Genomes, gnomAD, and ExAC databases were retained. TMB values were then normalized to the length of the coding sequence (CDS, captured regions for WES; whole genome for WGS). A TMB of ≥10 mutations per megabase (mut/Mb) was classified as TMB-High (TMB-H), whereas a TMB of <10 mut/Mb was classified as TMB-Low (TMB-L). Microsatellite instability (MSI) status was assessed using the MSIsensor2 software. Based on the software’s predefined criteria, an MSI score of 20% was used as the cutoff threshold: samples with an MSI score < 20% were classified as microsatellite stable (MSS), whereas those with a score ≥ 20% were classified as microsatellite instability-high (MSI-H).

### Drug screening assays

GC PDOs were digested into single cells and resuspended in Matrigel at a concentration of approximately 25,000 cells/mL. A 10 μL droplet was added to each well of a 96-well plate, resulting in a final density of 250 cells per well. After 4 days of seeding, the medium was replaced with drug-containing medium at physiological concentrations: 5-FU (Cmax in patients = 1.7–2.4 µM, range *in vitro* = 0.002 to 200 µM, MCE), Oxaliplatin (Cmax in patients = 3.8–10.1 µM, range *in vitro* = 0.01 to 1000 µM, MCE) [22], SN-38 (Cmax in patients is about 26 nM, range *in vitro* = 0.03 to 3000 nM, MCE), Epirubicin (Cmax in patients is 2 µM, range *in vitro* = 0.002 to 20 µM, MCE) [23], and Paclitaxel (Cmax in patients is 0.08 µM, range *in vitro* = 0.08 to 800 nM, MCE) [24]. Control samples were incubated with 0.1% dimethyl sulfoxide (DMSO, Beyotime Biotechnology, Shanghai, China). Following 4–6 days of drug treatment, the medium was removed, and cell viability was assessed using the MTS assay (Promega, Madison, WI, USA), with absorbance read at 490 nm on a microplate reader (A490). Relative viability was calculated using the following formula: Inhibition rate (IR, %) = 100 − (A490 drug-treated/ A490 vehicle-control) × 100 [25–27].

### Patients’ follow-up

Patients were followed up by telephone every 3 months to assess and document their general condition and examination results. The final follow-up date was July 30, 2024. DFS was defined as the period from surgery to tumor recurrence or metastasis [8]. Progression-free survival (PFS) was defined as the interval from randomization to the occurrence of progressive disease (PD) or death from any cause [28].

### Statistical analysis

All experiments were independently repeated three times. The tumor inhibition rate (IR) was calculated as: IR = (1 – mean experimental value/ mean control value) × 100%. Half-maximal inhibitory concentration (IC_50_) values were determined by non-linear regression using GraphPad Prism 7. Sensitivity ranking was determined by the ratio of the IC_50_ values of PDOs to the drug’s steady-state plasma concentration (Css), and samples were ranked accordingly. PDO sensitivity to chemotherapeutics was comprehensively evaluated by combining this ranking with the inhibition rate (IR) at the steady-state plasma concentration, where an IR ≥ 50% was considered indicative of sensitivity. Results are presented as mean ± standard deviation (SD). Statistical analyses were performed using SPSS 25.0. Comparisons between groups were made using Student’s t-test, Welch’s t-test, or one-way ANOVA, as appropriate. *P* value < 0.05 was considered statistically significant. Concordance between the *in vitro* drug sensitivity of GC PDOs and clinical efficacy was evaluated using IC_50_ values, IR, accuracy, sensitivity, and specificity. Kaplan-Meier analysis was used for survival analysis, and the log-rank test was used to determine statistical significance.

## Results

### Establishment of GC PDOs

**Fig 1:**
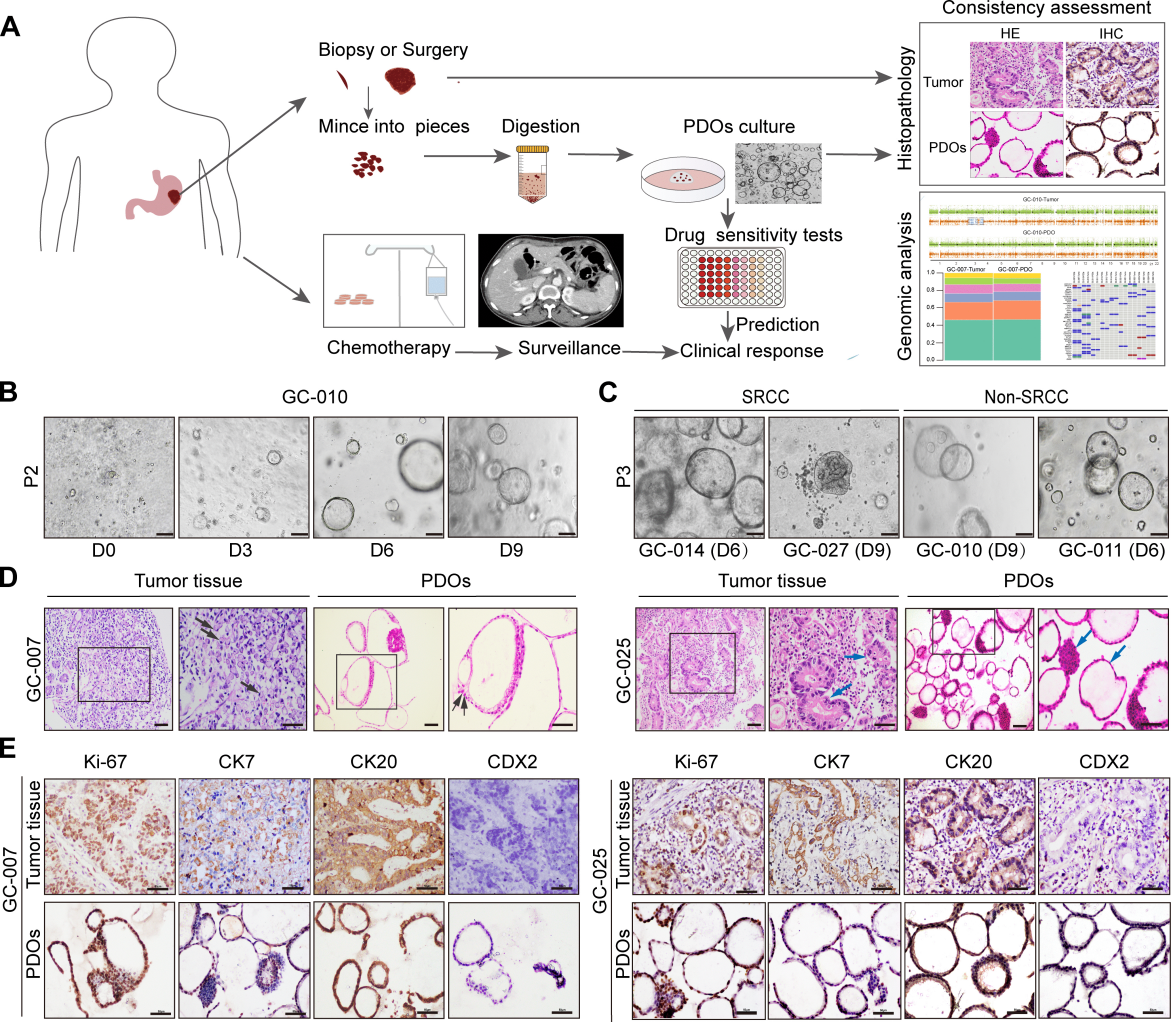
Study Workflow and histopathological characterization of GC PDOs. **(A)** Schematic overview of GC PDOs experimental workflow: tissue isolation, organoid culture, histopathological evaluation, genomic analysis, *in vitro* drug sensitivity testing, and prediction of clinical response. **(B)** Representative bright-field images of GC-010 PDOs from day 0 to day 9 post-seeding in Matrigel. Scale bars, 100 µm. Similar growth patterns were observed across all 17 established PDO lines. **(C)** Bright-field images highlighting the morphological diversity among PDOs derived from SRCC (GC-014, GC-027) and non-SRCC (GC-010, GC-011). Scale bars, 100 µm. **(D)** Representative H&E staining images of GC PDOs and their corresponding parental tumors. GC-007 is SRCC and GC-025 is non-SRCC. Black arrow indicates signet ring cells; blue arrow indicates solid or cystic structures. Scale bars, 50 µm. **(E)** IHC staining of Ki67, CK7, CK20 and CDX2 in GC PDOs and their parental tumors from GC-007 and GC-025. GC PDOs faithfully recapitulated histologic features of their parental tumors. Scale bars, 50 µm. P, passage. D, day. GC, gastric cancer. PDOs, patient-derived organoids. SRCC, signet-ring cell carcinoma. Non-SRCC, non-signet-ring cell carcinoma.

As shown in Table 1, a total of 27 GC samples from surgical resection or biopsy were collected for PDO establishment, and 17 PDOs were successfully generated at a rate of 63%. Among them, five samples contained signet-ring cell carcinoma (SRCC) components (accounting for 20–60%) (S1 Table in S1 File). No statistical significance was observed between clinicopathological characteristics (e.g., age, sex, TNM stage, and Lauren classification) and the success rate of PDO establishment (Table 1). Fig 1A illustrates the study workflow, including GC PDO construction and cultivation, *in vitro* drug sensitivity testing, and concordance analysis with clinical outcomes in matched patients. As shown in Fig 1B, the maximum diameter of individual PDOs reached approximately 100 µm after nine days of culture. Due to intratumoral heterogeneity, GC PDOs exhibited considerable morphological diversity, presenting solid, cystic, and mixed structures (S1A Fig in S2 File). Notably, no obvious morphological differences were observed between SRCC and non-SRCC PDOs, with the exception of GC-027, which displayed a dense structure (Fig 1C). The morphological features of PDOs remained stable over long-term passaging (S1B Fig in S2 File) and were preserved following cryopreservation and recovery (S1C Fig in S2 File).

**Table 1:** Relationship between patient’s clinicopathological features and the culture success rate of gastric cancer organoids.

Clinical features	Gastric cancer organoid culture		*P* value
	Number of successful cases	Success rate	
Gender			
Male	13	65%	*P* = 1.000
Female	4	57%	
Age (years)			
< 65	7	64%	*P* = 1.000
≥ 65	10	63%	
Tumor differentiation			
Moderate–high and moderate–low	10	71%	*P* = 0.440
Low	7	54%	
Lauren classification			
Intestinal	7	88%	*P* = 0.676
Mixed	8	73%	
Diffuse	2	25%	
Contains signet-ring cell			
Yes	5	63%	*P* = 1.000
No	12	63%	
TMN stage			
I-II	7	58%	*P* = 0.328
III	7	58%	
IV	3	100%	
Overall	17	63%	

*P* < 0.05 showed statistical difference.

### Histopathological and genomic characterizations of GC PDOs

H&E staining confirmed that the GC PDOs closely recapitulated the histological features of their corresponding primary tumors. These included disorganized glandular architecture (irregular mono- or multi-layered structures) and nuclear pleomorphism (variations in nuclear size, hyperchromatic, and prominent nucleoli). Notably, the GC-007 PDO (SRCC) reproduced the ring-like morphology of the parental tumor, reflecting the displacement of the nucleus to one side of the cell due to intracellular mucin accumulation. Similarly, the GC-025 PDO (non-SRCC) and its parental tumor both exhibited combined cystic and solid structures (Fig 1D). IHC staining for common GC markers showed positive immunoreactivity for the epithelial markers CK20 and CK7, as well as the proliferation marker Ki67, in both parental tumors and their corresponding PDOs. Furthermore, the CDX2 staining patterns in the PDOs were consistent with those in their parental tumors (Figs 1E and S2 in S2 File).

**Fig 2:**
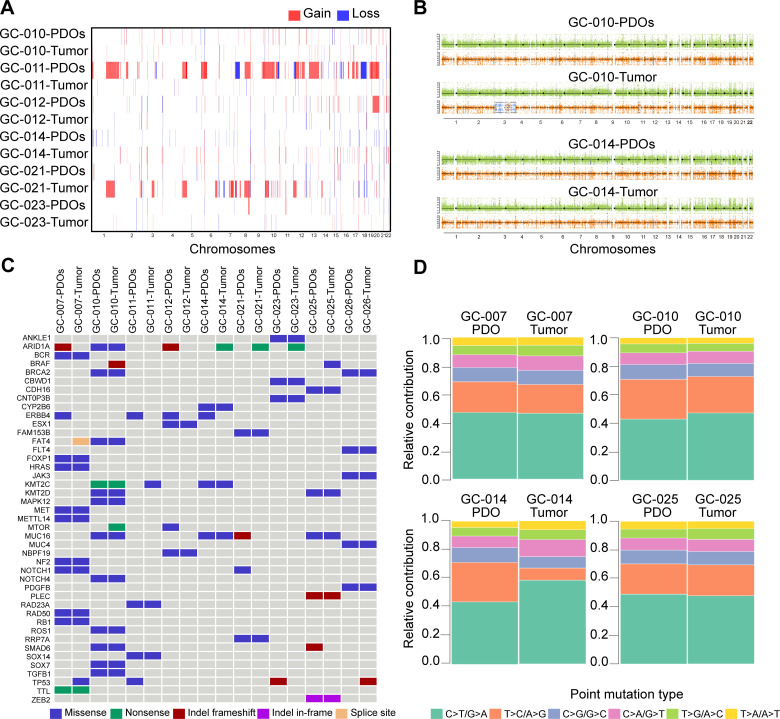
Genetic features of GC PDOs and their parental tumors. **(A)** Heatmap depicting CNVs in GC PDOs and their matched parental tumors. Columns represent individual samples, and rows represent genomic positions across chromosomes 1-22. The color scale indicates DNA copy number gains (red) and losses (blue). **(B)** Genome-wide CNVs profiles for two representative GC PDOs and their parental tumors (GC-010 and GC-014). The upper panels show genome-wide CNV profiles (chromosomes 1-22) with copy number gains (red), losses (blue), and regions with no change (green). The lower panels display B-allele frequency (BAF) distribution, showing balanced heterozygosity (orange), allelic imbalance (blue), and LOH events (BAF = 0/1). **(C)** Heatmap showing genetic alterations in the commonly mutated genes in GC, with alterations color-coded by type (e.g., missense, nonsense). **(D)** Distribution of base substitution types detected in GC PDOs and their corresponding parental tumor tissues (GC-007, GC-010, GC-014, GC-025). GC, gastric cancer. PDOs, patient-derived organoids.

We further verified the genetic consistency between GC PDOs and their corresponding primary tumors using WES. Most PDOs preserved the DNA CNV patterns observed in their parental tumors, including both gains and losses (Figs 2A, 2B and S3B in S2 File). Moreover, PDOs faithfully recapitulated common genomic alterations identified in the parental tumors, such as mutations in ARID1A, KMT2C, MUC16, BRCA2, TP53, FAT4 and RAD23A (Fig 2C). Six types of point mutations were identified: C > T/G > A, T > C/A > G, C > G/G > C, C > A/G > T, T > G/A > C and T > A/A > T, with C > T/G > A being the most frequent substitution. Furthermore, the mutational signature distribution was similar between PDOs and their parental tumors (Figs 2D and S3C in S2 File). Significantly mutated genes (SMGs) were identified via WES analysis, and the top 28 most frequency mutant genes included CMYA5 (33.33%), SVEP1 (33.33%), C10orf71 (22.22%), MAP1A (22.22%) and GSDMB (16.67%) (S3A Fig in S2 File). Comparison of TMB and MSI status between PDOs and their corresponding tumors revealed no statistically significant differences (TMB: *P* = 0.39, S3D Fig in S2 File; MSI: *P* = 0.73, S3E Fig in S2 File). Except for sample GC-010, which exhibited a TMB-H/MSI-H phenotype in the tumor tissue, all other samples were classified as TMB-L/MSS.

### Drug sensitivity analysis of GC PDOs to different chemotherapeutic agents

**Fig 3:**
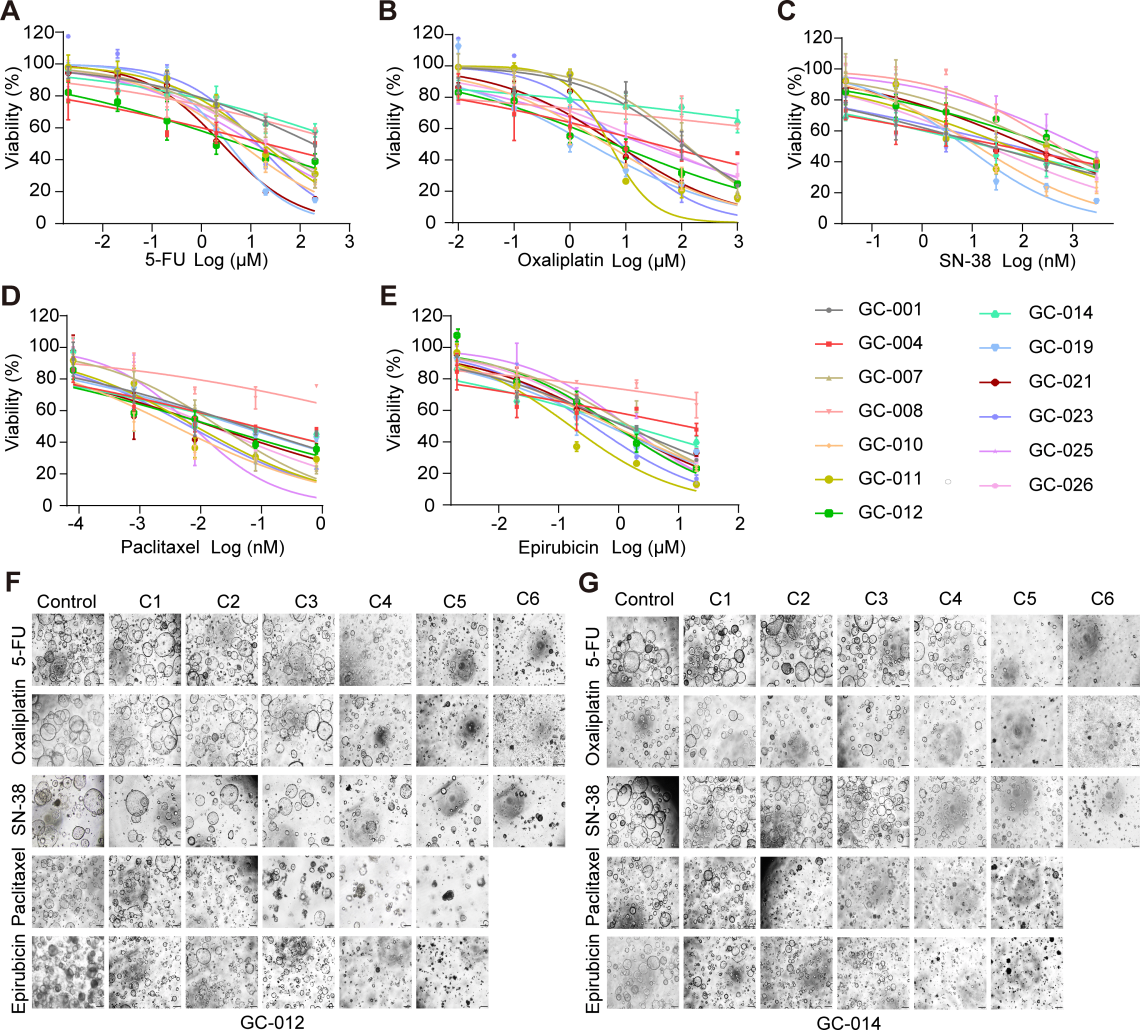
Response of GC PDOs to chemotherapeutic drugs. **(A-E)** Dose-response curves illustrating the sensitivity of PDOs to (A) 5-FU, (B) oxaliplatin, (C) SN-38, (D) paclitaxel, and (E) epirubicin. Data are presented as the mean ± SD from three biological replicates. Experiments were performed using 13 PDO lines. **(F-G)** Representative bright-field images of GC PDOs (GC-012, non-SRCC; GC-014, SRCC) treated with the indicated chemotherapeutic drugs at various concentrations. The tested concentrations were as follows: 5-FU (C1 = 0.002 µM, C2 = 0.02 µM, C3 = 0.2 µM, C4 = 2 µM, C5 = 20 µM, C6 = 200 µM), Oxaliplatin (C1 = 0.01 µM, C2 = 0.1 µM, C3 = 1 µM, C4 = 10 µM, C5 = 100 µM, C6 = 1000 µM), SN-38 (C1 = 0.03 nM, C2 = 0.3 nM, C3 = 3 nM, C4 = 30 nM, C5 = 300 nM, C6 = 3000 nM), Paclitaxel (C1 = 0.08 nM, C2 = 0.8 nM, C3 = 8 nM, C4 = 80 nM, C5 = 800 nM), Epirubicin (C1 = 0.002 µM, C2 = 0.02 µM, C3 = 0.2 µM, C4 = 2 µM, C5 = 20 µM). Scale bars, 100 µm. C, concentration. GC, gastric cancer. PDOs, patient-derived organoids. SRCC, signet-ring cell carcinoma. Non-SRCC, non-signet-ring cell carcinoma.

To evaluate the drug sensitivity of GC PDOs, five chemotherapeutic agents commonly used in GC treatment were tested. Dose-response curves revealed variable sensitivities of PDOs to different drugs, as well as substantial inter-PDO heterogeneity in drug responses (Fig 3A–3E). Moreover, drug sensitivity patterns were associated with specific pathological subtypes (SRCC or non-SRCC, S4 Fig in S2 File) and Lauren classification (S5 Fig in S2 File). PDOs derived from all SRCC samples and a subset of non-SRCC samples generally exhibited resistance to 5-FU and oxaliplatin, whereas some were sensitive to SN-38, paclitaxel and epirubicin (S2 and S3 Tables in S1 File). Representative bright-field images illustrated the dynamic growth characteristics of two PDO lines at different drug concentrations. PDOs from GC-012 (non-SRCC) exhibited a dose-dependent reduction in proliferation and an increased proportion of non-viable cells following treatment with 5-FU, oxaliplatin, paclitaxel, and epirubicin (Fig 3F). In contrast, PDOs from GC-014 (SRCC) showed a dose-dependent response only to SN-38, paclitaxel, and epirubicin (Fig 3G).

**Fig 4:**
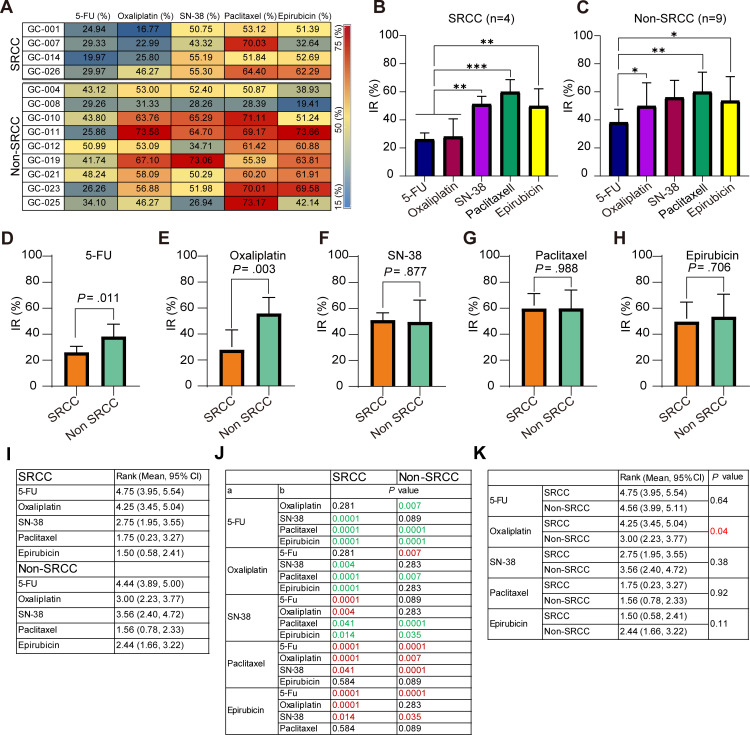
Sensitivity evaluation of GC PDOs to chemotherapeutic drugs. **(A)** Heatmap of tumor inhibition rates (IR) for 13 PDOs treated with five chemotherapeutic agents at steady-state plasma concentrations. **(B-C)** Comparison of the mean tumor IR among different chemotherapeutic drugs at steady-state plasma concentrations in (B) SRCC (n = 4) and (C) non-SRCC (n = 9) organoids. Data are presented as the mean ± SD. Statistical significance was determined using one-way ANOVA. *P < 0.05, **P < 0.01, ***P < 0.001. **(D-H)** Comparison of the mean tumor IR between PDOs from SRCC (n = 4) and non-SRCC (n = 9) for each chemotherapeutic drug. Data are presented as the mean ± SD. Statistical significance was determined using Welch’s *t-*test in (D) and Student’s *t*-test in (E-H). **(I)** Average sensi*t*ivity ranking and 95% CI of different chemotherapeutic drugs in SRCC (n = 4) and non-SRCC (n = 9). Sensitivity ranking was determined by the ratio of the IC_50_ values of PDOs to the drug’s steady-state plasma concentration, with lower values indicating higher sensitivity. **(J)** *P* values for the comparison of sensitivity rankings among chemotherapeutic drugs in SRCC and non-SRCC PDOs. Bold green font indicates that the average sensitivity ranking of drugs in column a is significantly lower than that in column b. Bold red font indicates that the average sensitivity ranking in column a is significantly higher than that in column b. Statistical significance was determined using one-way ANOVA. **(K)** Differences in the average sensitivity ranking and 95% CI between PDOs from SRCC and non-SRCC for each chemotherapeutic drug. Statistical significance was determined using Student’s *t*-test. *P* < 0.05 indica*t*es a statistically significant difference. n, number. GC, gastric cancer. PDOs, patient-derived organoids. IR, inhibition rate. SRCC, signet-ring cell carcinoma. Non-SRCC, non-signet-ring cell carcinoma. CI, confidence interval.

Tumor IRs were calculated to assess GC PDO responses to chemotherapeutic drugs at human plasma steady-state concentrations. Fig 4A shows that GC PDOs exhibited variable IRs to different chemotherapeutics, consistent with their dose-response curves. Compared with 5-FU and oxaliplatin, SN-38 (*P* = 0.002 and 0.003, respectively), paclitaxel (*P* = 0.0001 and 0.0001, respectively) and epirubicin (*P* = 0.003 and 0.005, respectively) demonstrated significantly greater tumor-inhibitory effects in SRCC. Among these, paclitaxel showed the strongest inhibition (IR = 59.85%) (Figs 4B, S6A and S6B in S2 File). In non-SRCC PDOs, oxaliplatin (*P* = 0.013), paclitaxel (*P* = 0.003), and epirubicin (*P* = 0.03) significantly reduced viability compared with 5-FU, with paclitaxel exhibiting the highest tumor IR (59.97%) (Figs 4C, S6A and S6B in S2 File). Furthermore, when comparing subtypes, non-SRCC PDOs were significantly more sensitive than SRCC PDOs to 5-FU (IR = 38.15%, *P* = 0.011) and oxaliplatin (IR = 55.90%, *P* = 0.003) (Figs 4D, 4E and S6C in S2 File). However, no statistically significant difference was observed between SRCC and non-SRCC PDOs in their response to SN-38, paclitaxel, or epirubicin (Figs 4F–H and S6C in S2 File).

In addition, sensitivity ranking of the chemotherapeutics was performed based on the ratio of IC_50_ to the human steady-state plasma drug concentration (S6D and S6E Fig in S2 File). In SRCC PDOs, both epirubicin (Rank = 1.5) and paclitaxel (Rank = 1.75) had significantly better ratios than 5-FU (*P* = 0.0001 for both), oxaliplatin (*P* = 0.0001 for both), and SN-38 (*P* = 0.014 and 0.041, respectively) (Figs 4I and 4J), with no significant difference between the top two ranked drugs (*P* = 0.584). In non-SRCC PDOs, paclitaxel and epirubicin demonstrated the highest average sensitivity rankings, with values of 1.56 and 2.44, respectively. Although paclitaxel ranked slightly higher, the difference was not statistically significant (*P* = 0.089) (Figs 4I and 4J). Furthermore, SRCC PDOs exhibited a significantly lower average sensitivity ranking compared to non-SRCC PDOs in the oxaliplatin-treated group (*P* = 0.04). However, no statistical difference was observed in 5-FU, SN-38, paclitaxel, or epirubicin treated group (Fig 4K).

### Consistency analysis of drug sensitivity of PDOs and clinical efficacy in matched GC patients

**Fig 5:**
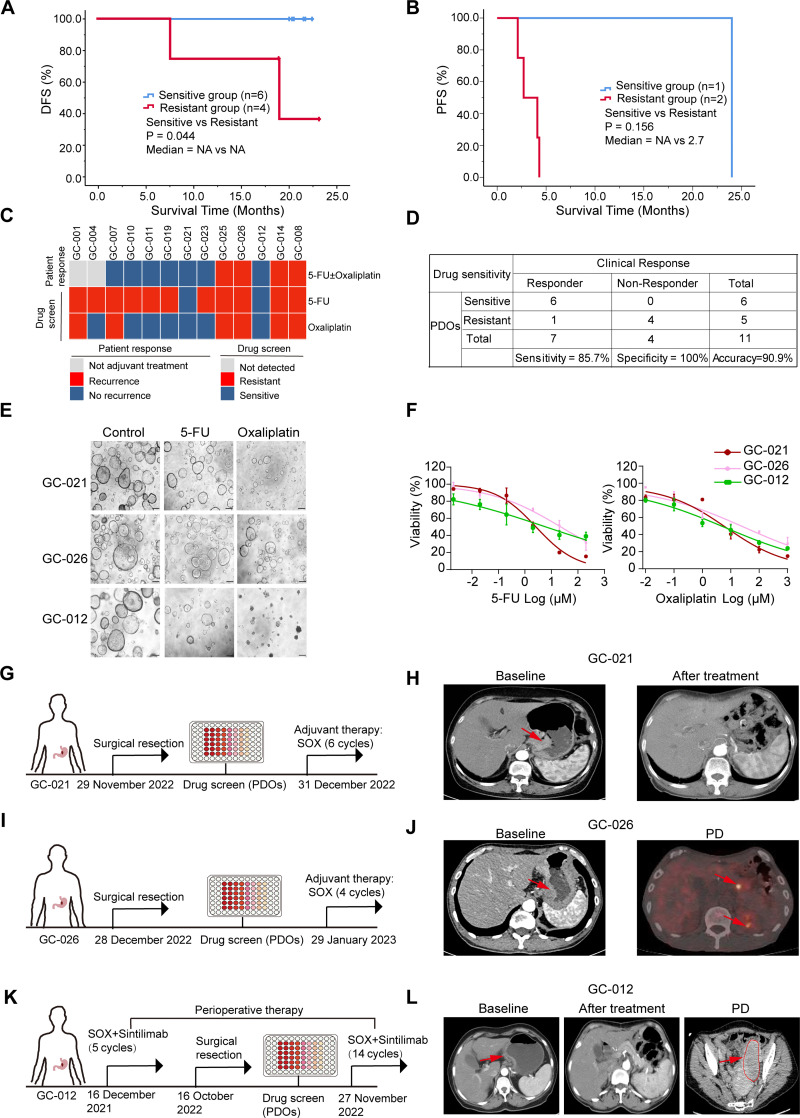
GC PDOs predict clinical outcomes of patients treated with chemotherapeutic agents. **(A)** Kaplan-Meier survival analysis showing DFS outcomes of the patients with matched PDOs. Based on the *in vitro* drug-sensitivity results of PDOs treated with the corresponding clinical adjuvant chemotherapy regimen, patients were divided into two groups: drug-sensitive (n  =  6) and drug-resistant (n  =  4). The *P* value was determined by log-rank test. **(B)** Kaplan-Meier survival analysis showing PFS outcomes of the patients with matched PDOs. Based on the *in vitro* drug-sensitivity results of PDOs treated with the corresponding clinical chemotherapy regimen, patients were divided into two groups: drug-sensitive (n  =  1) and drug-resistant (n  =  2). The *P* value was determined by log-rank test. **(C)** Heatmap summarizing the concordance between PDO drug screening results and patients’ clinical outcomes (13 patient cases). The heatmap displays patient outcomes and PDO responses to 5-FU and oxaliplatin. **(D)** Concordance analysis of drug sensitivity between clinical responses and matched GC PDOs samples. **(E)** Representative bright-field images of GC PDOs treated with vehicle, 5-FU and oxaliplatin in three selected cases. **(F)** Fitted dose-response curves of PDOs (GC-021, GC-026 and GC-012) representing the diverse patients’ responses to 5-FU and oxaliplatin. Data are presented as the mean  ±  SD from three biological replicates. **(G)** Clinical treatment timeline for patient GC-021 and corresponding drug screening results on matched PDOs. **(H)** CT scans of patient GC-021 at baseline and after postoperative adjuvant treatment. **(I)** Clinical treatment timeline for patient GC-026 and corresponding drug screening results on matched PDOs. **(J)** CT scans of patient GC-026 at baseline and at the metastasis stages. **(K)** Clinical treatment timeline for patient GC-012 and corresponding drug screening results on matched PDOs. **(L)** CT scans of patient GC-012 at baseline and after postoperative adjuvant treatment. Red arrows indicate tumors. GC, gastric cancer. PDOs, patient-derived organoids. DFS, disease-free survival. PFS, progression-free survival. NA, not reached. PD, progressive disease.

As shown in S4 Table in S1 File, most patients received standard chemotherapy regimens combining S-1 and oxaliplatin (SOX). Patients GC-001 and GC-004 did not receive postoperative therapy for personal reasons. Among patients who underwent radical surgery followed by adjuvant chemotherapy, those whose PDOs exhibited sensitivity to at least one chemotherapeutic agent *in vitro* had a median follow-up duration of 21.0 months (interquartile range [IQR]: 20.3–21.6), during which no tumor recurrence was observed. In contrast, patients whose PDOs were resistant to all tested drugs had a median follow-up of 18.8 (IQR: 15.8–20.2) months, and 2 patients experienced tumor recurrence. Kaplan-Meier survival analysis showed significantly better DFS for patients in the PDO drug-sensitive group compared with the drug-resistant group (*P* = 0.044) (Fig 5A). Among patients with stage IV GC, those with drug-resistant PDOs had a median PFS of only 2.7 months (95% CI: 0.74–4.66). In contrast, patients with PDOs that exhibited *in vitro* sensitivity to at least one tested drug achieved a markedly prolonged PFS of 24 months (Fig 5B). The pharmacophenotyping results of the 13 PDOs and their matched patients’ clinical outcomes are summarized in Fig 5C. As shown in Fig 5D, the drug sensitivity of GC PDOs *in vitro* was highly correlated with patients’ clinical outcomes, with sensitivity, specificity and accuracy values of 85.7%, 100% and 90.9%, respectively.

In postoperative patients, PDOs derived from GC-010, GC-011, GC-019, GC-021, and GC-023 were sensitive to 5-FU and oxaliplatin, which closely aligned with the corresponding patients’ favorable clinical response to these agents (Figs 5E–5H and S7A–S7C in S2 File and S4 Table in S1 File). PDOs from GC-025 and GC-026 (SRCC) were resistant to 5-FU and oxaliplatin, and the matched patient developed intraperitoneal lymph node or duodenal metastasis after postoperative therapy (Figs 5E, 5F, 5I, 5J and S7C in S2 File).

Among the stage IV patients, GC-012 was diagnosed with GC and right ovarian metastasis and received neoadjuvant SOX combined with sintilimab, followed by total gastrectomy and right adnexectomy. According to the Mandard’s classification system (tumor regression grade, TRG), the pathological response to neoadjuvant therapy was excellent (TRG 1), indicating only microscopic residual disease (tumor foci <0.1 cm in diameter). Postoperatively, the patient received an additional 14 cycles of treatment and achieved a PFS of 24 months, after which recurrence was detected in the left adnexa. Consistent with the clinical efficiency, the matched PDOs were sensitive to both 5-FU (IR = 50.99%), and oxaliplatin (IR = 53.09%) (Figs 5E, 5F, 5K and 5L and S4 Table in S1 File). Patient GC-008, diagnosed with advanced GC, received three cycles of neoadjuvant therapy with SOX and camrelizumab followed by radical surgery, but subsequently developed liver metastases (PFS1: 123 days). Subsequent treatments included paclitaxel and capecitabine, followed by irinotecan combined with apatinib and tislelizumab, each yielding short PFS (PFS2: 130 days, PFS3: 80 days). Liver metastases were biopsied and corresponding PDOs were established for drug screening. Consistent with the clinical course, PDOs-based *in vitro* drug sensitivity testing showed complete resistance to 5-FU (IR = 29.26%), oxaliplatin (IR = 31.33%), SN-38 (IR = 28.26%), and paclitaxel (IR = 28.39%) (S8A Fig in S2 File and S4 Table in S1 File). Additionally, patient GC-014, who received SOX combined with sintilimab as first-line treatment, achieved a PFS of only 62 days. Corresponding PDOs demonstrated resistance to 5-FU (IR = 19.97%), oxaliplatin (IR = 25.80%) (S8B Fig in S2 File and S4 Table in S1 File).

## Discussion

Standard, uniform adjuvant chemotherapy regimens for locally advanced GC, including specific subtypes such as SRCC, yield suboptimal efficacy, with persistently low 5-year survival rate [2]. This underscores the urgent need for personalized treatment strategies to address the profound intertumoral heterogeneity in therapeutic responses. PDO-based drug screening has emerged as a promising approach to advance precision oncology by tailoring therapies to the individual tumor [14,15]. In this study, we successfully established PDOs from patients with locally advanced GC and systematically evaluated the concordance between PDO drug responses and clinical outcomes. Our findings demonstrate the feasibility and clinical relevance of PDO-guided adjuvant therapy in enhancing treatment efficacy and improving patient survival.

Drug sensitivity testing revealed that individual PDO lines exhibited variable responses to different chemotherapeutic agents, and a single drug often displayed distinct tumor inhibition rates across different PDOs. This pronounced inter-PDO heterogeneity underscores the limitations of applying uniform regimens to a biologically diverse patient population, particularly given the divergent drug responses observed across GC pathological subtypes. Notably, PDOs derived from SRCC or diffuse-type GC were more sensitive to epirubicin and paclitaxel but resistant to 5-FU and oxaliplatin, in contrast to non-SRCC PDOs. Chen et al. reported that a taxane-based regimen demonstrated superior efficacy in the treatment of gastric SRCC [29]. The MATCH study reported that perioperative chemotherapy with docetaxel, oxaliplatin and S-1 significantly improved the major pathological response and tended to yield better PFS compared with SOX in locally advanced GC [30]. Additionally, Yu et al. found that in stage III GC patients who underwent radical D2 gastrectomy, adjuvant chemotherapy with nab-paclitaxel plus S-1 significantly improved both DFS and OS compared with the standard oxaliplatin and capecitabine combination [31]. Collectively, these findings highlight the need for randomized controlled trials (RCTs) to compare postoperative treatment efficacy between SRCC and non-SRCC, and to identify the optimal chemotherapy regimen for each pathological subtype. Furthermore, our results strongly support the initiation of clinical trials evaluating adjuvant therapies containing paclitaxel and/or epirubicin versus the conventional 5-FU and oxaliplatin combination in patients with SRCC. Such investigations may help establish more effective and personalized treatment strategies, thereby potentially improving DFS in patients with locally advanced, operable GC.

Our findings underscore the potential value of PDOs in precision treatment of GC patients. The accuracy, sensitivity and specificity of PDOs in predicting responses to different chemotherapeutics were 90.9%, 85.7% and 100%, respectively, which are comparable to those reported in colorectal cancer [22] and lung cancer [32]. For patients who received cognate adjuvant treatments, no tumor recurrence was detected in those with drug-sensitive PDOs, while half of the patients with drug-resistant PDOs experienced tumor recurrence. A significant difference in DFS was observed between the two groups, despite the limited median DFS due to the small sample size. For advanced GC, the PDOs drug screening system demonstrates clear advantages. Specifically, the stage IV GC patient (GC-012) with PDOs sensitive to the SOX regimen responded well clinically, achieving a PFS of 24 months, which surpasses the median PFS reported for many first-line treatments combining of immunotherapy and chemotherapy [33,34]. In contrast, the median PFS of stage IV patients (GC-008, GC-014) whose PDOs were resistant to 5-FU and oxaliplatin was only 2.7 months. Patient GC-008, whose PDOs were resistant to multiple chemotherapeutics, experienced rapid disease progression and a poor prognosis despite radical gastrectomy and subsequent lines of therapy. Collectively, these findings highlight a strong concordance between PDO-derived drug sensitivity profiles and clinical responses, validating PDOs as a robust preclinical model for personalized efficacy prediction in locally advanced or metastatic GC. While immunotherapy has transformed oncology, the rational selection of chemotherapeutic agents remains pivotal for optimizing outcomes in advanced GC. Ryul et al. reported that chemotherapy-resistant tumors exhibited fewer effector T cells and a higher abundance of tumor-associated macrophages, potentially leading to both chemotherapy resistance and diminished efficacy of immunotherapy [35]. This highlights the importance of conducting *in vitro* chemosensitivity screening using PDO models, even in the era of cancer immunotherapy.

In this study, we demonstrated that GC PDOs closely recapitulate the histopathological and genetic characteristics of the parental tumors. However, some discrepancies in the mutation spectrum were observed, mainly due to the complex cellular composition of parental tumor tissue versus the higher purity of PDOs. Minor discrepancies in TMB and MSI between each organoid and its parent tumor tissue primarily arose from several factors: the loss of the tumor microenvironment during culture; selective culture conditions enriched specific subclones, potentially altering TMB/MSI representation; and tumor spatial heterogeneity, which means that the small biopsy used to establish organoids might not fully represent the entire tumor, thereby amplifying sampling bias. In addition, GC PDOs were successfully established with a success rate of 63%. The reasons for the establishment failure include contamination, tissue hardness, and tumor necrosis. Notably, three SRCC-derived PDOs ceased to proliferate due to tight binding with stromal fibroblasts. Additionally, the dormant phase of signet-ring cells requires elaborate culture conditions to prevent cell death [36]. Therefore, the success rate of PDO establishment could be improved by optimizing tissue handling procedures and refining culture conditions.

This study has several limitations. Firstly, the relatively small sample size may reduce the predictive power, warranting validation in a large cohort. Secondly, the PDO model was established from limited tumor tissue samples. During prolonged culture, PDOs progressively lose tissue-specific cell types, including niche-specific mesenchyme, immune cells, vascularization, innervation, and the microbiome. This loss restricts the PDO model’s capacity to fully recapitulate tumor heterogeneity. Given the critical role of heterogeneity in mediating drug resistance, future efforts should focus on establishing PDOs from multi-region biopsies to better capture intratumoral diversity.

## Conclusions

In conclusion, PDOs of locally advanced GC faithfully recapitulate the histopathological and genetic features of their parental tumors and accurately mirror clinical responses to chemotherapy. Our data reveal that specific pathological subtypes, such as SRCC and diffuse-type GC, are resistant to 5-FU and oxaliplatin but sensitive to paclitaxel and epirubicin. This suggests that conventional adjuvant chemotherapy regimens for GC may not be optimal for patients with SRCC or diffuse-type GC, highlighting the need for alternative therapeutic strategies. In summary, our findings demonstrate that PDOs can reliably predict patient responses in the clinic and hold significant potential for guiding precision medicine in GC.

## Supporting information

S1 Data Source data. (XLSX)

S1 File Supplementary Tables S1-S4. (PDF)

S2 File Supplementary Figures S1-S8. (PDF)
