# Supplementary material for: Patient-derived organoids predict chemotherapy response of locally advanced gastric cancer
Source: PLoS One. 2026 Mar 9;21(3):e0339416. doi: 10.1371/journal.pone.0339416 (PMC12970873; doi:10.1371/journal.pone.0339416)
Supplement: S1 File — (PDF) [file pone.0339416.s002.pdf]

S1 Table. Clinicopathological characteristics of gastric cancer patients with successfully established PDOs

ID	Pathology
GC-001	(Stomach) moderately-poorly differentiated adenocarcinoma with signet-ring cell carcinoma (20%)
GC-004	(Stomach) moderately-poorly differentiated adenocarcinoma
GC-007	(Stomach) poorly differentiated adenocarcinoma with signet-ring cell carcinoma (40%)
GC-008	Liver metastases originated from poorly differentiated adenocarcinoma of the stomach
GC-010	(Stomach) moderately differentiated adenocarcinoma
GC-011	(Stomach) moderately-poorly differentiated adenocarcinoma
GC-012	(Stomach) specimen after neoadjuvant therapy, poorly differentiated adenocarcinoma
GC-014	(Stomach) moderately-poorly differentiated adenocarcinoma with signet-ring cell carcinoma (40%)
GC-015	(Stomach) high-moderately differentiated adenocarcinoma
GC-019	(Stomach) moderately-poorly differentiated adenocarcinoma
GC-020	(Stomach) poorly differentiated adenocarcinoma
GC-021	(Stomach) poorly differentiated adenocarcinoma
GC-023	(Stomach) moderately-poorly differentiated adenocarcinoma
GC-024	(Stomach) moderately differentiated adenocarcinoma
GC-025	(Stomach) moderately-poorly differentiated adenocarcinoma
GC-026	(Stomach) poorly differentiated adenocarcinoma with signet-ring cell carcinoma (45%)
GC-027	(Stomach) poorly differentiated adenocarcinoma with signet-ring cell carcinoma (60%)

S2 Table. IC_{50} values of SRCC-derived organoids against various chemotherapeutic drugs

SRCC	GC-001	GC-007	GC-014	GC-026
5-FU(μM)				
IC_{50}	183.4±0.16	21.58±0.11	1127±0.35	22.46±0.1
95% CI	94.19-442.2	12.91-37.11	292.7-10213	14.17-37.19
Oxaliplatin(μM)				
IC_{50}	104.9±0.12	117.2±0.09	1653762±1.46	30.76±0.13
95% CI	58.74-186.8	76.23-181	17029-5303313795785	16.88-57.69
SN-38(nM)				
IC_{50}	23.03±0.18	293.9±0.20	20.92±0.40	20.05±0.38
95% CI	10.02-57.82	115.2-929.3	3.02-264	7.65-58.75
Paclitaxel(nM)				
IC_{50}	0.06±0.28	0.02022±0.08	0.07194±0.38	0.01554±0.22
95% CI	0.016-0.34	0.014-0.03	0.012-1.31	0.0051-0.055
Epirubicin(μM)				
IC_{50}	1.237±0.09	1.543±0.16	1.581±0.13	0.8345±0.11
95% CI	0.80-1.93	0.72-3.49	0.87-3.14	0.48-1.46

SRCC: signet ring cell carcinoma; IC_{50}: (half-maximal inhibitory concentrations); CI: confidence intervals.

S3 Table. IC_{50} values of non-SRCC organoids against various chemotherapeutic drugs

Non-SRCC	GC-004	GC-008	GC-010	GC-011	GC-012	GC-019	GC-021	GC-023	GC-025
5-FU (μM)									
IC_{50}	20.63±0.42	972.2±0.33	6.17±0.078	17.62±0.08	6.12±0.17	3.46±0.06	2.61±0.07	13.52±0.15	12.21±0.09
95% CI	3.46-483.4	276.6-6933	4.24-9.02	11.92-26.43	2.77-14.51	2.65-4.52	1.84-3.67	6.41-31.44	7.87-19.49
Oxaliplatin (μM)									
IC_{50}	35.33±0.74	699718±1.16	4.63±10.1	5.16±0.12	6.26±0.09	2.25±0.17	6.79±0.98	8.25±0.15	21.96±0.17
95% CI	6.72-501.3	15717-15314894586	2.9-7.4	2.91-10.98	4.07-9.67	1.01-4.9	4.15-10.69	3.87-18.03	10.01-52.2
SN-38 (nM)									
IC_{50}	45.36±0.29	528±0.11	12.61±0.13	55.82±0.32	445.3±0.17	7.37±0.1	144.6±0.19	74.73±0.31	1543±0.13
95% CI	11.44-232.2	306.1-958.3	6.83-23.38	12.28-449.3	213.2-1069	4.56-11.84	59.95-416.7	17.53-458.2	883.6-3104
Paclitaxel (nM)									
IC_{50}	0.078±0.37	31.98±1.04	0.004±0.17	0.008±0.16	0.018±0.15	0.047±0.19	0.016±0.29	0.007±0.19	0.0073±0.16
95% CI	0.016-1.46	1.536-1422442	0.002-0.008	0.004-0.02	0.008-0.04	0.02-0.14	0.004-0.079	0.002-0.019	0.003-0.02
Epirubicin (μM)									
IC_{50}	13.32±0.49	7692±1.04	0.84±0.095	0.1591±0.097	0.87±0.088	0.68±0.13	0.84±0.1	0.38±0.064	1.43±0.11
95% CI	1.98-1521	260.5-199636452	0.53-1.36	0.10-0.26	0.57-1.34	0.37-1.3	0.51-1.48	0.28-0.56	0.82-2.52

Non-SRCC, Non signet ring cell carcinoma; IC_{50}, half-maximal inhibitory concentration; CI: confidence intervals

S4 Table. Clinical characteristics and drug sensitivity of patient-derived gastric cancer organoids

ID	TNM stage	Therapies	PDO response（IR, %）	Drug sensitivity	DFS/PFS（M）	Clinical Response
GC-001	II	No	5-FU/Oxaliplatin (29.94/16.77)	Resistance	23.3	NO recurrence
GC-004	II	No	5-FU/Oxaliplatin (43.12/**53.00**)	Sensitivity	22.4	NO recurrence
GC-007	III	SOX (Adjuvant treatment)	5-FU/Oxaliplatin (29.33/22.99)	Resistance	18.5	NO recurrence
GC-010	III	SOX (Adjuvant treatment)	5-FU/Oxaliplatin (43.80/**63.76**)	Sensitivity	21.7	NO recurrence
GC-011	II	SOX (Adjuvant treatment)	5-FU/Oxaliplatin (25.86/**73.58**)	Sensitivity	21.5	NO recurrence
GC-019	III	Oxaliplatin (Adjuvant treatment)	Oxaliplatin (**67.10**)	Sensitivity	20.5	NO recurrence
GC-021	II	SOX (Adjuvant treatment)	5-FU/Oxaliplatin (48.24/**58.09**)	Sensitivity	20.3	NO recurrence
GC-023	III	SOX (Adjuvant treatment)	5-FU/Oxaliplatin (26.26/**56.88**)	Sensitivity	20.0	NO recurrence
GC-025	III	SOX (Adjuvant treatment)	5-FU/Oxaliplatin (34.10/46.27)	Resistance	19.1	Recurrence
GC-026	III	SOX (Adjuvant treatment)	5-FU/Oxaliplatin (29.27/46.27)	Resistance	7.7	Recurrence
GC-012	IV	SOX plus sintilimab (Perioperative treatment)	5-FU/Oxaliplatin (**50.99**/**53.09**)	Sensitivity	24.0	Recurrence
GC-014	IV	SOX plus sintilimab (First-line treatment)	5-FU/Oxaliplatin (19.97/25.80)	Resistance	2.1	Progressive disease
		SOX plus camrelizumab (neoadjuvant treatment)	5-FU/Oxaliplatin (29.26/31.33)	Resistance	4.1	
GC-008	IV	Paclitaxel plus capecitabine (Second-line treatment)	5-FU/Paclitaxel (29.26/28.39)	Resistance	4.1	Progressive disease
		Irinotecan plus apatinib and tislelizumab (third-line treatment)	Irinotecan (28.26)	Resistance	2.7	

IR, inhibition rate; DFS, disease free survival; PFS, progression free survival, SOX, S-1 combined with oxaliplatin; Underline('_'), resistance.
