# Supplementary material for: Patient-derived organoids predict chemotherapy response of locally advanced gastric cancer
Source: PLoS One. 2026 Mar 9;21(3):e0339416. doi: 10.1371/journal.pone.0339416 (PMC12970873; doi:10.1371/journal.pone.0339416)
Supplement: S2 File — (PDF) [file pone.0339416.s003.pdf]

Supplementary Figures

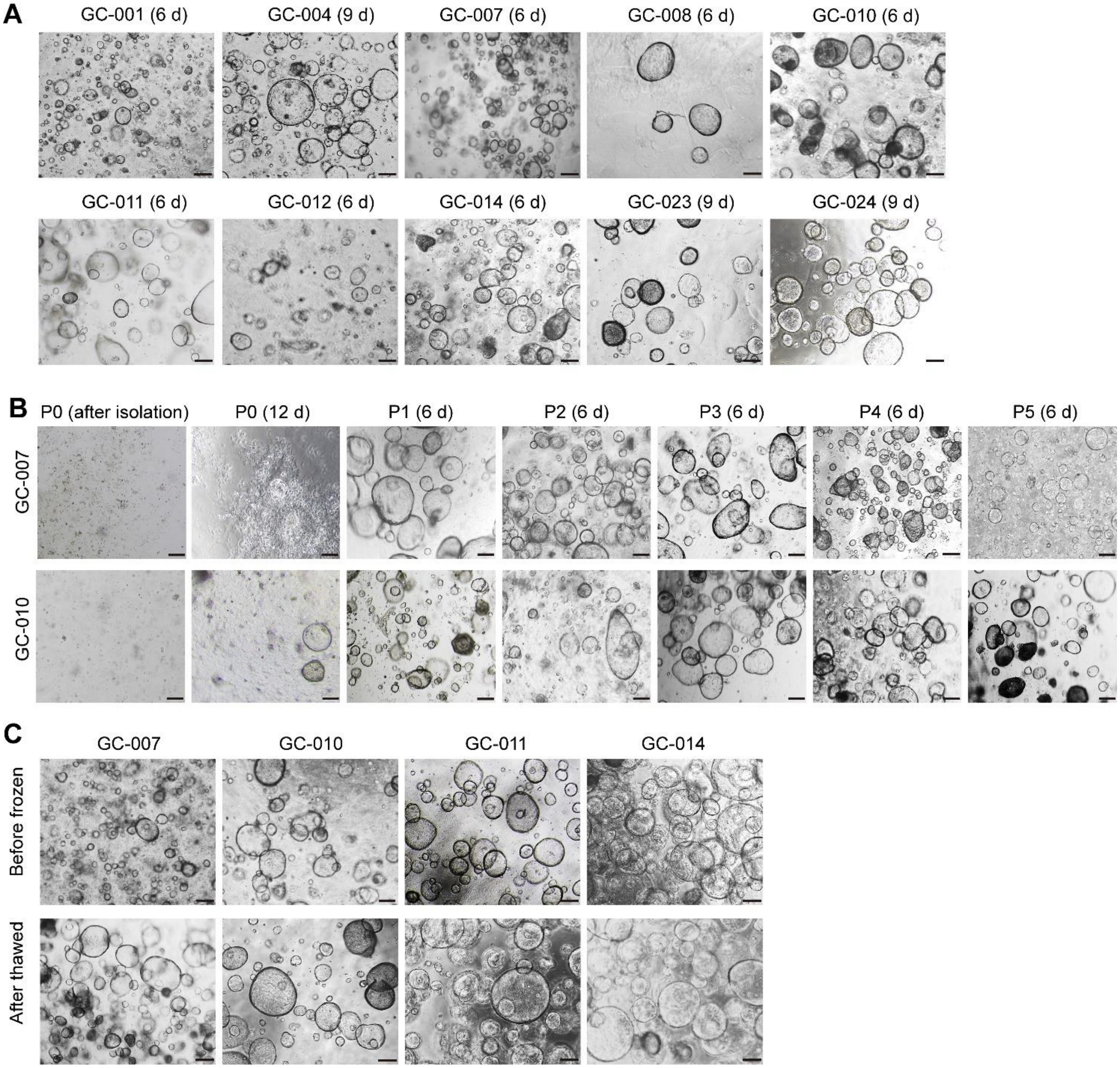

S1 Fig. Bright field performance of GC PDOs. (A) Bright-field images showing the morphology of representative GC PDOs. (B) Morphological features of two representative PDOs (GC-007 and GC-010) during isolation, culture and passage. (C) Bright-field images of cryopreservation and recovery of GC PDOs. GC-007 and GC-014 are SRCC, GC-010 and GC-011 are non-SRCC. Scale bars, 100 μm. GC, gastric cancer. PDOs, patient-derived organoids. SRCC, signet-ring cell carcinoma. Non-SRCC, non-signet-ring cell carcinoma. P, passage. d, day.

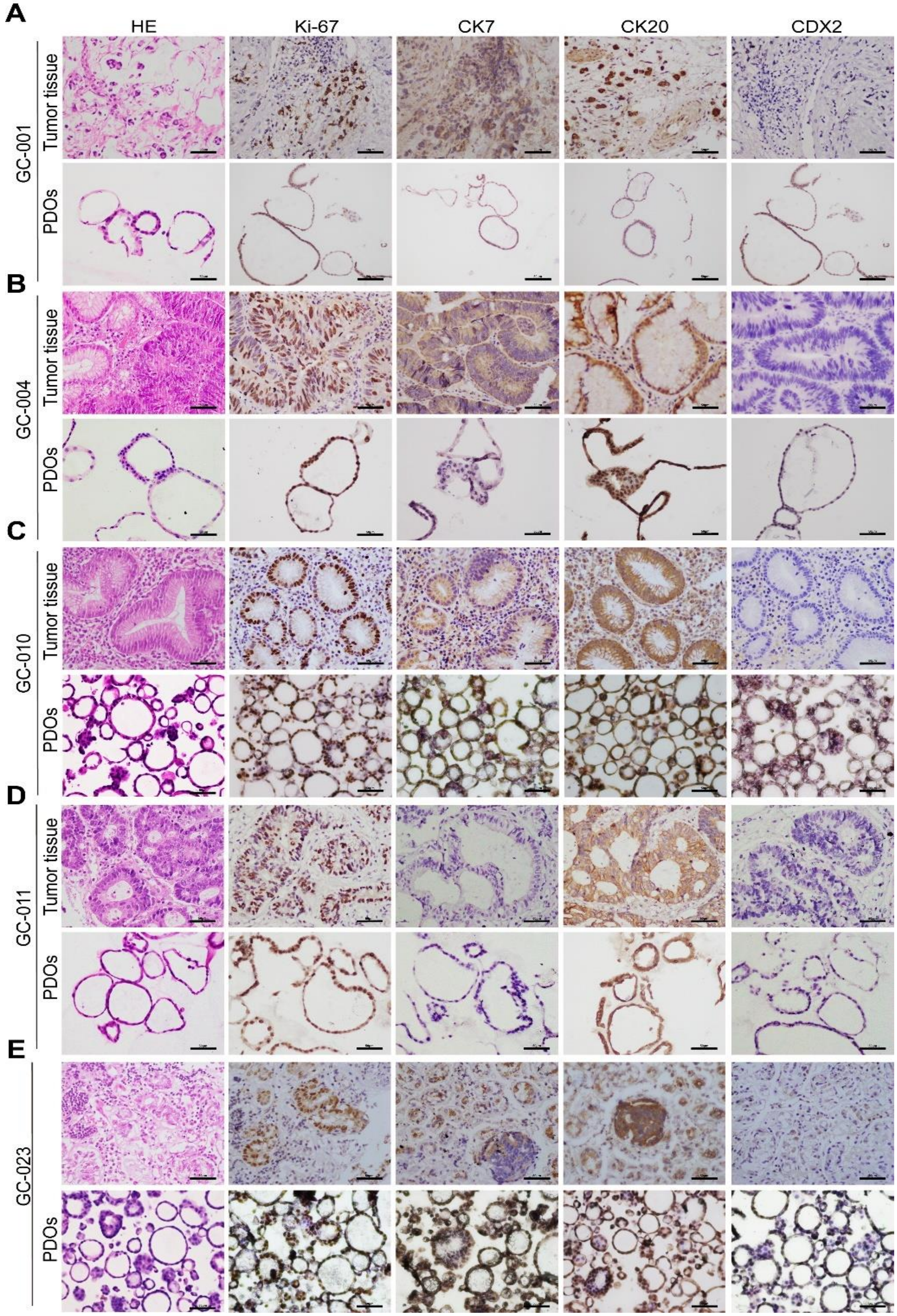
A
HE
Ki-67
CK7
CK20
CDX2
GC-001
Tumor tissue
PDOs
B
GC-004
Tumor tissue
PDOs
C
GC-010
Tumor tissue
PDOs
D
GC-011
Tumor tissue
PDOs
E
GC-023
PDOs

S2 Fig. H&E and IHC staining images of GC PDOs and the parental tumors. (A-E) H&E and IHC staining images of PDOs from GC-001, GC-004, GC-010, GC-011, GC-023, and their parental tumors, respectively. GC PDOs recapitulated morphological and histological features of their parental tumors. GC, gastric cancer. PDOs, patient-derived organoids.

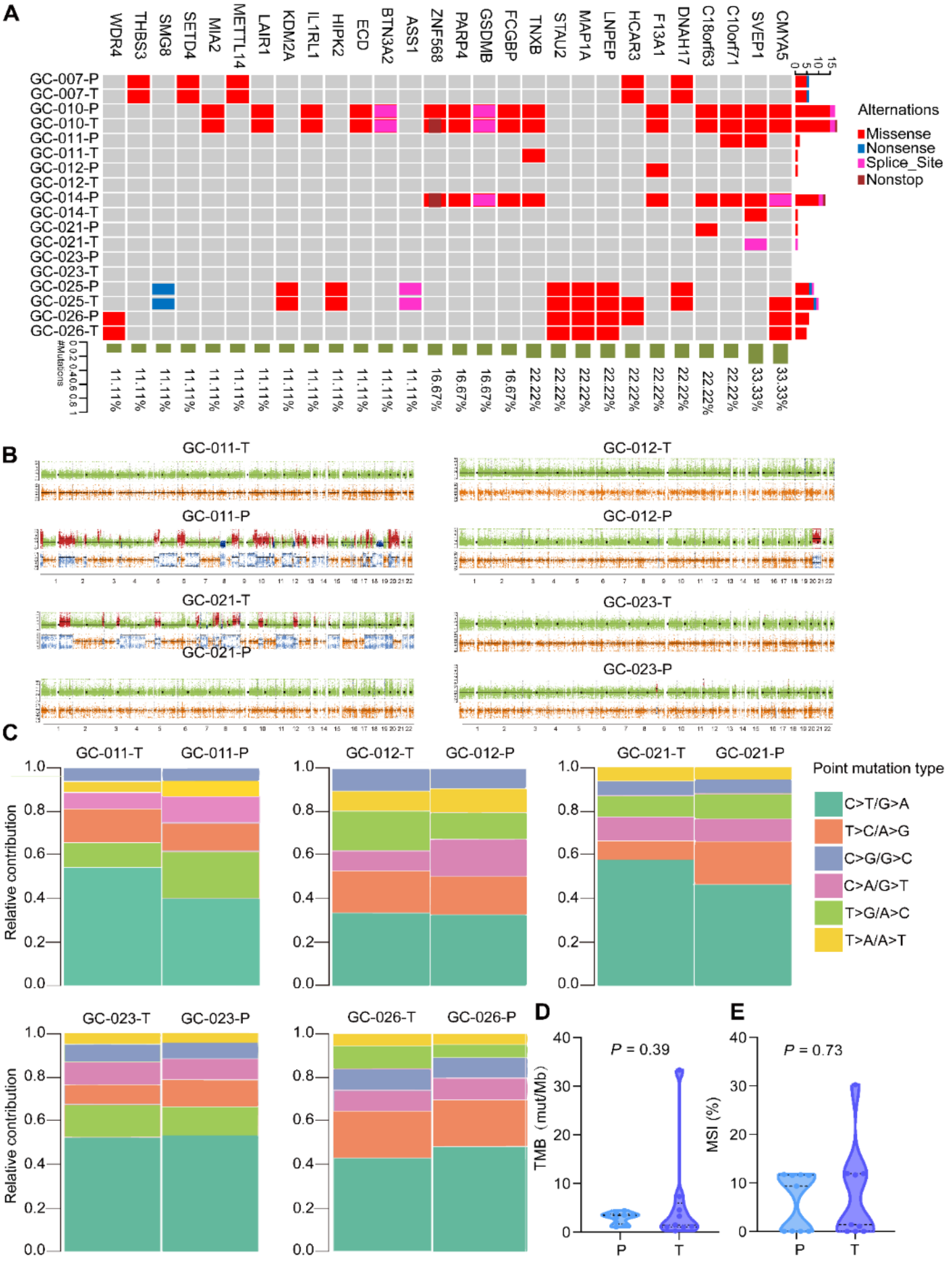

S3 Fig. Analysis of genetic alternations in GC PDOs. (A) Overview of the top 28 significantly mutated genes (SMGs), demonstrating the mutational concordance between PDOs and their matched parental tumors. (B) Genome-wide CNVs of representative GC PDOs and their parental tumors (GC-

011, GC-012, GC-021, GC-023; blue, gains; red, losses; green, no change). The upper panel shows genome-wide CNV profiles (chromosomes 1-22) with copy number gain (red), lose (blue), and neutral regions (green). The lower panel displays B-allele frequency (BAF) distribution showing balanced heterozygosity (orange), allelic imbalance (blue), and LOH events (BAF=0/1). (C) Bar graph displaying the different contributions of the point mutation types for five pairs of GC PDOs and their parental tumors. (D) Tumor mutational burden (TMB) in PDOs versus matched parental tumors. Statistical significance was determined using Student's t-test. (E) Comparison of microsatellite instability (MSI) status between PDOs and their matched parental tumors. Statistical significance was determined using Student's t-test. GC, gastric cancer. T, tumor. P, patient-derived organoids. CNVs, copy number variations.

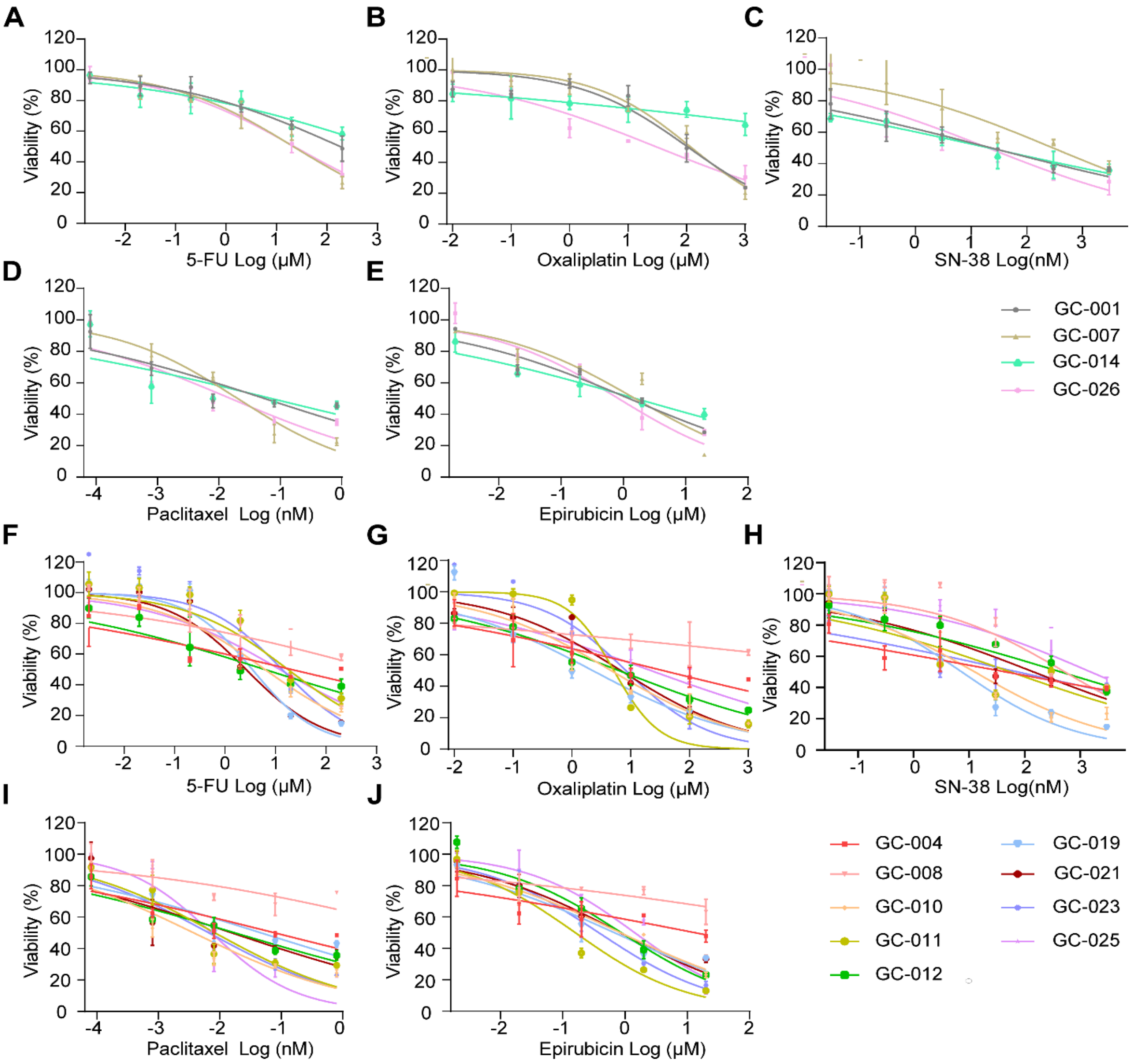

S4 Fig. Dose-response curves of PDOs from SRCC and non-SRCC to different anti-GC chemotherapeutic drugs. (A-E) Dose-response curves of SRCC PDOs (n=4) to: 5-FU, oxaliplatin, SN-38, paclitaxel, and epirubicin. (F-J) Dose-response curves of non-SRCC PDOs (n=9) to: 5-FU, oxaliplatin, SN-38, paclitaxel and epirubicin. Data are presented as the mean ± SD from three biological replicates. GC, gastric cancer. PDOs, patient-derived organoids. SRCC, signet-ring cell carcinoma. Non-SRCC, non-signet-ring cell carcinoma.

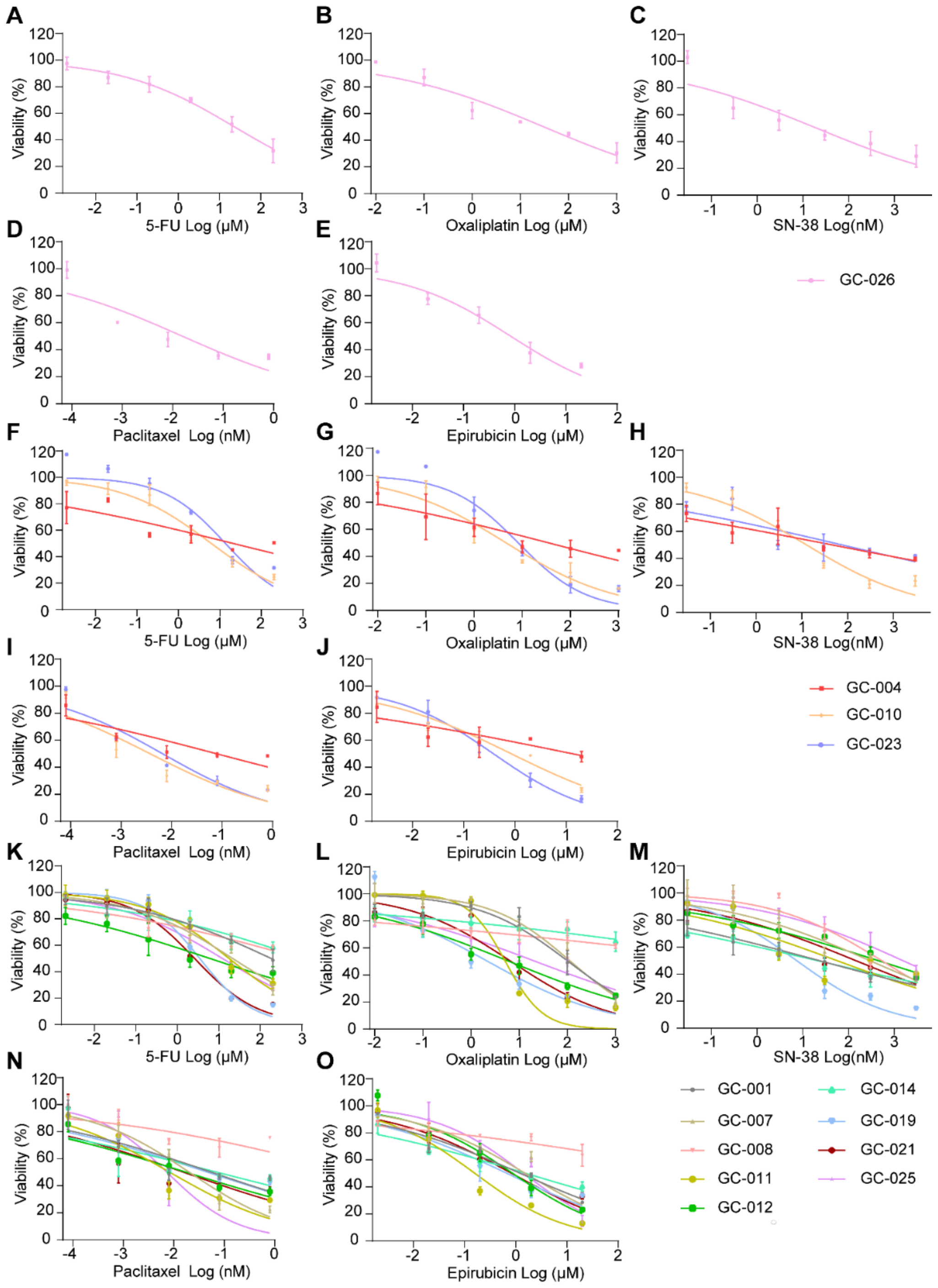

S5 Fig. Dose-response curves of GC PDOs from Lauren's classification of GC to different anti-GC chemotherapeutic drugs. (A-E) Dose-response curves of PDOs from diffuse type (n=1) to: 5-FU, oxaliplatin, SN-38, paclitaxel and epirubicin. (F-J) Dose-response curves of PDOs from intestinal type (n=3) to: 5-FU, oxaliplatin, SN-38, paclitaxel and epirubicin. (K-O) Dose-response curves of PDOs

from mixed type (n=9) to: 5-FU, oxaliplatin, SN-38, paclitaxel and epirubicin. Data are presented as the mean ± SD from three biological replicates. GC, gastric cancer. PDOs, patient-derived organoids.

A

SRCC	IR (%, Mean and 95% CI)
5-FU	26.05 (18.68, 33.42)
Oxaliplatin	27.96 (7.62, 48.29)
SN-38	51.14 (42.18, 60.09)
Paclitaxel	59.85 (45.80, 73.89)
Epirubicin	49.75 (30.02, 69.48)
Non-SRCC	
5-FU	38.15 (30.84, 45.46)
Oxaliplatin	55.90 (46.41, 65.39)
SN-38	49.75 (36.92, 62.58)
Paclitaxel	59.97 (49.14, 70.80)
Epirubicin	53.51 (40.18, 66.83)

B

		SRCC	Non-SRCC
a	b	*P* value	
5-FU	Oxaliplatin	0.78	0.013
	SN-38	0.002	0.117
	Paclitaxel	0.0001	0.003
	Epirubicin	0.003	0.03
Oxaliplatin	5-Fu	0.78	0.013
	SN-38	0.003	0.361
	Paclitaxel	0.0001	0.554
	Epirubicin	0.005	0.727
SN-38	5-Fu	0.002	0.117
	Oxaliplatin	0.003	0.361
	Paclitaxel	0.213	0.141
	Epirubicin	0.839	0.563
Paclitaxel	5-Fu	0.0001	0.003
	Oxaliplatin	0.0001	0.554
	SN-38	0.213	0.141
	Epirubicin	0.152	0.349
Epirubicin	5-Fu	0.003	0.03
	Oxaliplatin	0.005	0.727
	SN-38	0.839	0.563
	Paclitaxel	0.152	0.349

C

		IR (%, Mean and 95% CI)	*P* value
5-FU	SRCC	26.05 (18.68, 33.42)	0.011
	Non-SRCC	38.15 (30.84, 45.46)	
Oxaliplatin	SRCC	27.96 (7.62, 48.29)	0.003
	Non-SRCC	55.90 (46.41, 65.39)	
SN-38	SRCC	51.14 (42.18, 60.09)	0.877
	Non-SRCC	49.75 (36.92, 62.58)	
Paclitaxel	SRCC	59.85 (45.80, 73.89)	0.988
	Non-SRCC	59.97 (49.14, 70.80)	
Epirubicin	SRCC	49.75 (30.02, 69.48)	0.706
	Non-SRCC	53.51 (40.18, 66.83)	

D

	Drug	5-FU	Oxaliplatin	SN-38	Paclitaxel	Epirubicin
SRCC	GC-001	76.42	10.39	0.89	0.70	0.62
	GC-007	563.50	11.60	11.30	0.25	0.77
	GC-014	11.23	163738.81	0.80	0.90	0.79
	GC-026	10.79	3.05	0.77	0.19	0.42
Non-SRCC	GC-004	10.32	3.50	1.74	0.98	6.66
	GC-008	486.10	69279.01	20.31	399.75	3846.00
	GC-010	3.09	0.46	0.49	0.05	0.42
	GC-011	8.81	0.51	2.15	0.10	0.08
	GC-012	3.06	0.62	17.13	0.22	0.44
	GC-019	1.73	0.22	0.28	0.58	0.34
	GC-021	1.30	0.67	5.56	0.20	0.42
	GC-023	6.76	0.82	2.87	0.08	0.19
	GC-025	6.11	2.17	59.35	0.09	0.72

E

	Drug	5-FU	Oxaliplatin	SN-38	Paclitaxel	Epirubicin
SRCC	GC-001	5	4	3	2	1
	GC-007	5	4	3	1	2
	GC-014	4	5	2	3	1
	GC-026	5	4	3	1	2
Non-SRCC	GC-004	5	3	2	1	4
	GC-008	3	5	1	2	4
	GC-010	5	3	4	1	2
	GC-011	5	3	4	2	1
	GC-012	4	3	5	1	2
	GC-019	5	1	2	4	3
	GC-021	4	3	5	1	2
	GC-023	5	3	4	1	2
	GC-025	4	3	5	1	2

S6 Fig. Sensitivity evaluation of PDOs from SRCC and non-SRCC to different anti-GC chemotherapeutic drugs. (A) Average tumor IR and 95% CI of different anti-GC chemotherapeutic drugs in PDOs from both SRCC and non-SRCC. (B) *P* values of tumor IR for drugs in PDOs from both SRCC and non-SRCC. Bold green font indicates that the average tumor IR of drugs in column a is lower than in column b. Bold red font indicates that the average tumor IR of drugs in column a is higher than that in column b. Statistical significance was determined using one-way ANOVA. (C) Difference in average tumor IR and 95% CI between PDOs from SRCC and non-SRCC for different anti-GC chemotherapeutic drugs, respectively. Statistical significance was determined using Welch's *t*-test (5-FU) or Student's *t*-test (other drugs). (D) The ratio of sensitivity ranking was calculated by IC_{50}/the steady-state plasma concentration of the drug. (E) Sensitivity ranking sorted according to the ratio in Figure S6D, the smaller ratio, the higher sensitivity ranking. $P < 0.05$ shows significant statistic differences. GC, gastric cancer. PDOs, patient-derived organoids. SRCC, signet-ring cell carcinoma. Non-SRCC, non-signet-ring cell carcinoma. IR, inhibition rate. CI, confidence interval.

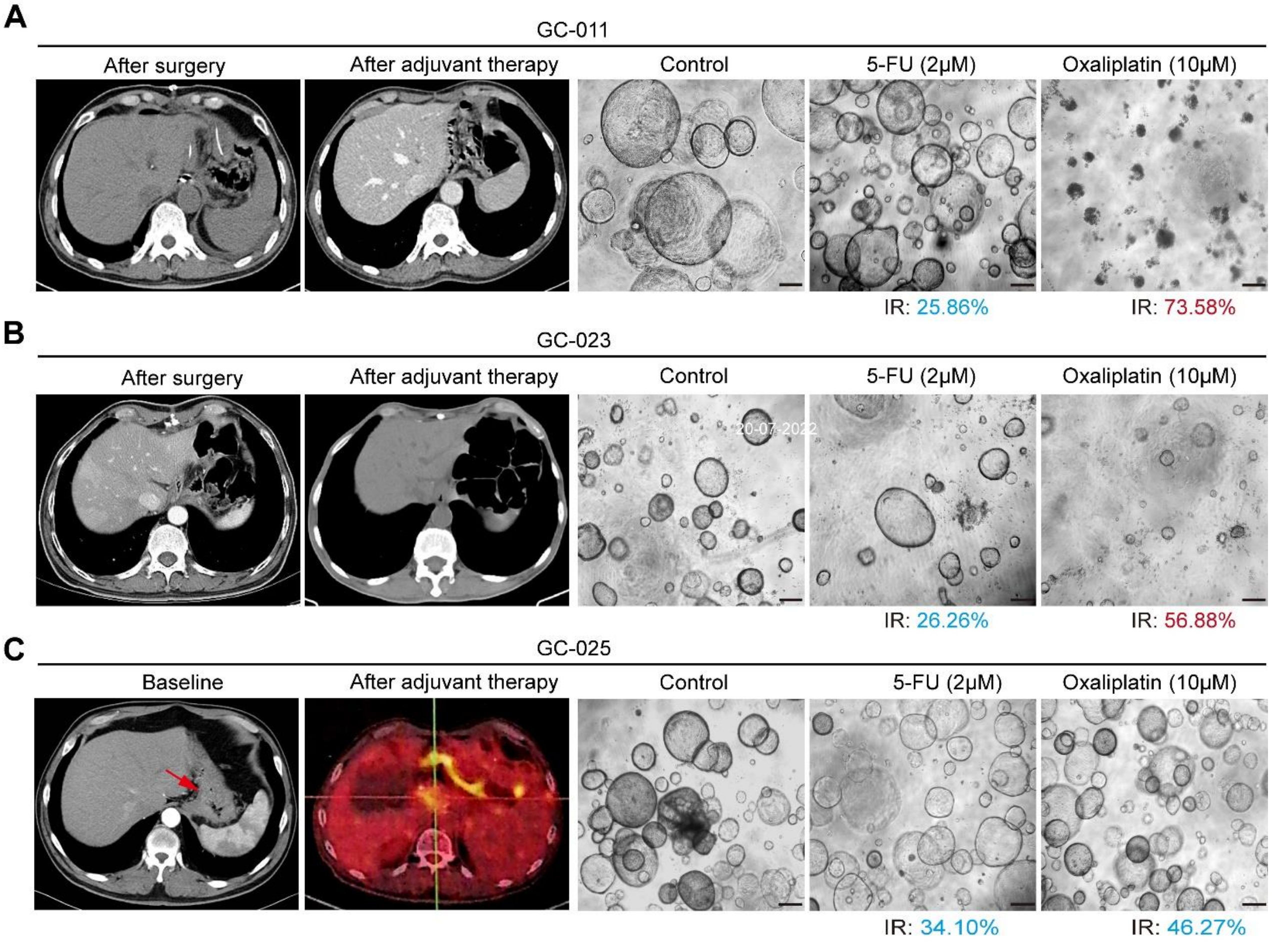

S7 Fig. Consistency evaluation of drug sensitivity of PDOs and clinical response in corresponding patients with postoperative GC. (A-B) CT scans of patients with GC-011 and GC-023 before and after postoperative adjuvant treatment are shown on the left side; bright-field images and tumor IR of PDOs treated with vehicle, 5-FU, and oxaliplatin are shown on the right side. Red font, sensitive. Blue font, resistant. (C) CT scans of patient with GC-025 before and after postoperative adjuvant treatment are shown on the left side; bright-field images and tumor IR of PDOs treated with vehicle, 5-FU, and oxaliplatin are shown on the right side. Blue font, resistant. The red arrows indicate tumors. GC, gastric cancer. PDOs, patient-derived organoids. IR, inhibition rate.

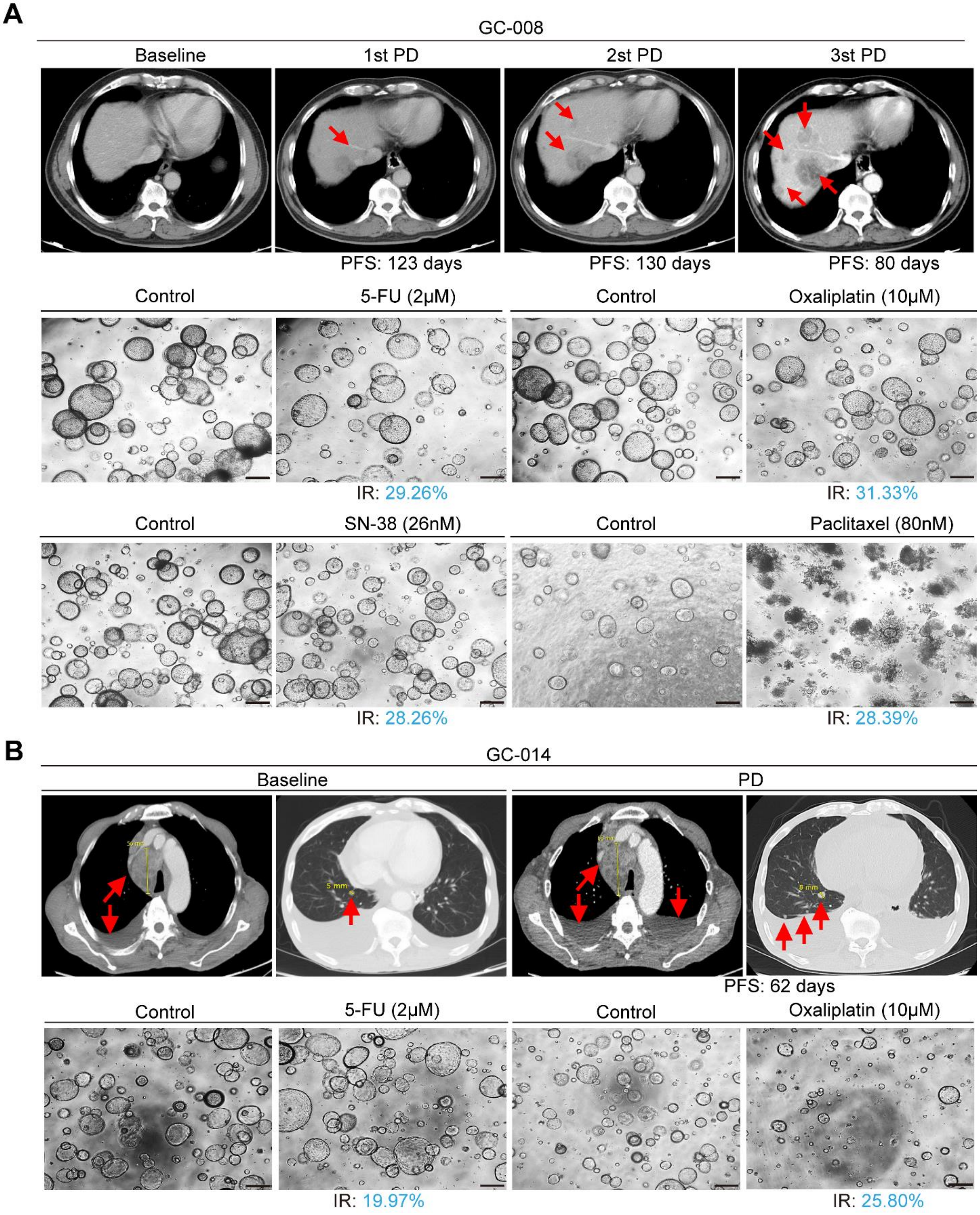

S8 Fig. Consistency evaluation of drug sensitivity of PDOs and clinical response in corresponding patients with stage IV GC. (A) CT scans of patient with GC-008 at the baseline and PD stages are

shown on the left side; bright-field images and tumor IR of PDOs treated with vehicle, 5-FU, oxaliplatin, SN-38 and paclitaxel are shown on the right side. Blue font, resistant. (B) CT scans of patient with GC-014 at the baseline and PD stages are shown on the left side; bright-field images and tumor IR of PDOs treated with vehicle, 5-FU, and oxaliplatin are shown on the right side. Blue font, resistant. The red arrows indicate tumors. GC, gastric cancer. PDO, patient-derived organoids. PD, progressive disease. IR, inhibition rate.
